# Medroxyprogesterone improves nocturnal breathing in postmenopausal women with chronic obstructive pulmonary disease

**DOI:** 10.1186/1465-9921-6-28

**Published:** 2005-04-04

**Authors:** Tarja Saaresranta, Tero Aittokallio, Karri Utriainen, Olli Polo

**Affiliations:** 1 Sleep Research Unit at the Department of Physiology, University of Turku, Sleep Research Unit, Dentalia, Lemminkäisenkatu 2, 20520 Turku, Finland; 2 Department of Pulmonary Diseases, Turku University Central Hospital, 20520 Turku, Finland; 3 Department of Mathematics, University of Turku, 20014 Turku, Finland; 4 Department of Pulmonary Diseases, Tampere University Central Hospital, P.O.Box 2000, 33521 Tampere, Finland

## Abstract

**Background:**

Progestins as respiratory stimulants in chronic obstructive pulmonary disease (COPD) have been investigated in males and during wakefulness. However, sleep and gender may influence therapeutic responses. We investigated the effects of a 2-week medroxyprogesterone acetate (MPA) therapy on sleep and nocturnal breathing in postmenopausal women.

**Methods:**

A single-blind placebo-controlled trial was performed in 15 postmenopausal women with moderate to severe COPD. A 12-week trial included 2-week treatment periods with placebo and MPA (60 mg/d/14 days). All patients underwent a polysomnography with monitoring of SaO_2 _and transcutaneous PCO_2 _(tcCO_2_) at baseline, with placebo, with medroxyprogesterone acetate (MPA 60 mg/d/14 days), and three and six weeks after cessation of MPA.

**Results:**

Thirteen patients completed the trial. At baseline, the average ± SD of SaO_2 _mean was 90.6 ± 3.2 % and the median of SaO_2 _nadir 84.8 % (interquartile range, IQR 6.1). MPA improved them by 1.7 ± 1.6 %-units (95 % confidence interval (CI) 0.56, 2.8) and by 3.9 %-units (IQR 4.9; 95% CI 0.24, 10.2), respectively. The average of tcCO_2 _median was 6.0 ± 0.9 kPa and decreased with MPA by 0.9 ± 0.5 kPa (95% CI -1.3, -0.54). MPA improved SaO_2 _nadir and tcCO_2 _median also during REM sleep. Three weeks after cessation of MPA, the SaO_2 _mean remained 1.4 ± 1.8 %-units higher than at baseline, the difference being not significant (95% CI -0.03, 2.8). SaO_2 _nadir was 2.7 %-units (IQR 4.9; 95% CI 0.06, 18.7) higher than at baseline. Increases in SaO_2 _mean and SaO_2 _nadir during sleep with MPA were inversely associated with baseline SaO_2 _mean (r = -0.70, p = 0.032) and baseline SaO_2 _nadir (r = -0.77, p = 0.008), respectively. Treatment response in SaO_2 _mean, SaO_2 _nadir and tcCO_2 _levels did not associate with pack-years smoked, age, BMI, spirometric results or sleep variables.

**Conclusion:**

MPA-induced respiratory improvement in postmenopausal women seems to be consistent and prolonged. The improvement was greater in patients with lower baseline SaO_2 _values. Long-term studies in females are warranted.

## Background

Chronic obstructive pulmonary disease (COPD), consisting of variable degrees of pulmonary emphysema and chronic obstructive bronchitis, has a male predominance. However, the prevalence of COPD is steadily increasing among women [1] as a consequence of increased rates of cigarette smoking. Women may be more sensitive to the deleterious effects of smoking than men[2]. Mortality due to COPD is increasing among women,[3] which may not merely reflect the increasing overall prevalence, but also the increase of the most severe forms of COPD. Therefore, novel therapeutic options are needed to cope with COPD.

Decreased production of progestins after menopause may result in lack of natural respiratory stimulation and predispose women to respiratory insufficiency. Therefore, progestin replacement therapy could effectively improve ventilation and oxygenation during sleep.

Hypoxemia and hypercapnia during sleep herald respiratory insufficiency. Many studies have investigated effects of progestins on breathing in awake subjects but few studies have evaluated the effect of progestin treatment on nocturnal breathing in patients with respiratory insufficiency, mostly due to COPD [4-9]. In light of the previous observations, the effect of progestin therapy on breathing may be weaker during sleep[5]. A key issue is that the aforementioned studies have recruited almost entirely men. However, effects of progestin therapy may be gender-dependent. We have previously reported of sustained progestin-induced improvement in awake blood gases of postmenopausal women with respiratory insufficiency[10]. We hypothesised that the beneficial effects of medroxyprogesterone acetate (MPA) would also be demonstrated as improved SaO_2 _and tcCO_2 _during sleep. We therefore wanted to evaluate the degree and duration of MPA effect on sleep and nocturnal breathing in postmenopausal women with chronic respiratory disease and show that MPA effectively improves SaO_2 _and tcCO_2 _during sleep in postmenopausal women with moderate to severe COPD.

## Methods

### Patient selection

15 consecutive postmenopausal women with COPD fulfilling the inclusion criteria and willing to volunteer were recruited for the trial by using our hospital data base. The inclusion criteria were forced expiratory volume in one second (FEV1) less than 65 % of predicted value[11]. The criteria for postmenopausal status were age over 50 yr, time since last menstruation at least 12 months, and serum concentrations of follicle-stimulating hormone (FSH) > 30 IU/l. All patients were receiving optimal therapy and had been in a stable clinical condition for at least a month prior the study.

The exclusion criteria included malignancies, heart diseases (except cor pulmonale), insulin dependent diabetes mellitus, previous thromboembolic events, obstructive sleep apnea syndrome, untreated hypothyroidism, any other major diseases, current smoking, long-term oxygen therapy and use of systemic postmenopausal hormone therapy. Patients were on their regular medication that was found optimal by their pulmonologist. Medication varied from patient to patient and included both short- and long-acting inhaled beta-agonists, anticholinergics and inhaled corticosteroids. No changes in medication were allowed throughout the study period. Medication likely to influence sleep or vigilance such as benzodiazepines, psychostimulants, melatonin or antidepressants were not allowed. Vaginal estrogen therapy was allowed since vaginal application does not affect serum estradiol levels.

All patients gave their written informed consents. The study protocol was approved by the Joint Commission on Ethics of Turku University and Turku University Central Hospital.

### Study design

**Figure 1:**
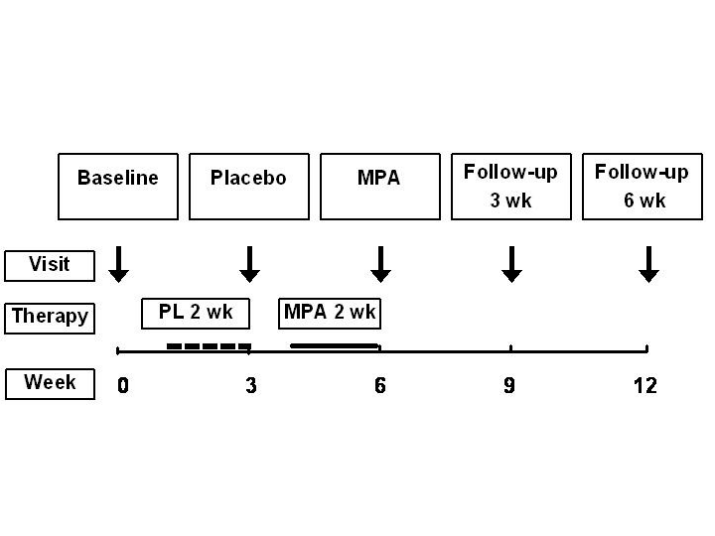
**Study design. **Both placebo (PL) and medroxyprogesterone acetate (MPA) periods lasted for 14 days. Arrows indicate the timing of sleep studies, dashed line the timing and duration of the placebo period and solid line thetiming and duration of the MPA period.

The 12-week study followed a placebo-controlled single-blind design (Fig. 1) and included five nights at 3-week intervals in sleep laboratory. Seven days after the baseline measurements, all patients started with placebo treatment for 14 days. After a 7-day interval, MPA treatment for 14 days was started. Patients were then followed-up for six weeks after cessation of MPA. We found it important that the placebo night data would be obtained before the patients had received MPA. Based on our earlier observations,[10,12,13] MPA-induced effects are measurable for weeks after cessation of progestin therapy. To exclude the carry-over effect, more than a 6-week washout period would be needed between the treatments. Knowing that acute exacerbations are frequent in patients with moderate to severe COPD, the longer duration of the study would increase the drop-out rate markedly. Therefore, the placebo and MPA studies were not done in a random sequence.

30 mg of oral MPA (Lutopolar^®^, Orion Pharma, Espoo, Finland) or placebo tablets provided by the same pharmaceutical company were administered twice in the evening, at 9 PM and 11 PM. Peak serum concentrations are reached within one to three hours followed by a sharp decline thereafter[14]. MPA was divided in two doses given two hours apart to minimize the variation in MPA levels during night. Compliance was assessed by tablet counts, patient interviews and measurements of serum MPA concentrations. The overnight polysomnography was registered five times at 3-week intervals: at baseline, after 14 days on placebo, after 14 days on MPA, and after 3- and 6-week washout periods. Sleep studies were performed at a university sleep laboratory, where the technician was present during the recording. In the evening, patients were weighed, and at each visit blood pressure was measured by the same researcher, using the auscultatory method in seated subjects. Every morning after sleep study patients completed a structured questionnaire with 14 separate items concerning their subjective sleep quality during the past night[15]. Questions were related to initiation and maintenance of sleep (for example, perceptions of sleep latency, frequency of awakenings), events during sleep (for example, restlessness), sleep-related daytime symptoms (for example, naps during the preceding day), and issues possibly disturbing sleep (for example, dyspnoea, sweating). Flow-volume spirometry (Vitalograph Compact II, Vitalograph Ltd, Buckingham, England) was assessed in the morning after the sleep study.

An overnight polysomnography included electroencephalogram, electro-oculogram, and chin electromyogram. Arterial oxyhemoglobin saturation (SaO_2_) was measured with pulse oximeter using a finger probe (Ohmeda Biox 3700 Pulse Oximeter, BOC Health Care, U.S.A.). The response time setting for the pulse oximeter was 6 seconds. Partial pressure of carbon dioxide (tcCO_2_) was measured transcutaneously (TINA^® ^Transcutaneous pO_2_/pCO_2 _Monitoring System, Radiometer, Copenhagen, Denmark). After cleansing the skin with alcohol the skin sensor was placed on the upper part of the chest parasternally and heated up to 43.5°C, at which temperature the sensor could remain attached continuously for 8 hours. Body movements were monitored with a static-charge-sensitive bed[16,17]. Quantitative movement analysis and SaO_2 _analyses were performed with custom made analysis modules (UniPlot^®^, Unesta, Turku, Finland). Nasal pressure was recorded with nasal prongs connected to a presser transducer. The nasal pressure signal was analysed with Sullivan Autoset device (Sullivan Autoset^®^, ResCare Limited, Sydney, Australia) used in a diagnostic mode and with a separate inspiratory flow shape analysis software described earlier[18]. Biosignals were recorded and stored with sampling frequency of 250 Hz per channel and with y-resolution of 12 bytes (UniPlot^®^, Unesta, Turku, Finland). The episodes of arterial oxyhemoglobin desaturation of 4 %-units or more per hour (ODI_4_) were calculated with Uniplot^® ^software (UniPlot^®^, Unesta, Turku, Finland). EtCO_2 _and tcCO_2 _signals were recorded with a sampling frequency of 100 Hz throughout the night by Embla^® ^system (Embla^®^, Flaga, Reykjavik, Iceland). The SaO_2 _and tcCO_2 _data were analyzed on a breath-by-breath basis. Each breath was assigned with the sleep stage during which it occurred. This was achieved using an MS-Excel macro, which combined the information from the two files according to time tags. The maximum inspiratory slope during the initial part of inspiration was determined on a breath-by-breath basis from the nasal pressure signal and used as an index of respiratory drive as previously described[19,20].

Sleep stages and arousals were scored according to standard criteria[21,22] and expressed as percentage of total sleep time. Sleep efficiency was defined as percentage sleep during the sleep period time.

### Statistical analyses

The overall comparisons between repeated measurements were performed with either nonparametric Friedman's test or parametric analysis of variance (ANOVA) for repeated measurements. In nonparametric case Wilcoxon signed ranks test and in parametric case F-test were used. In repeated measurements, Bonferroni correction was used for p-values. All comparisons were made to baseline measurements. Comparisons between the first and the second sessions tested the placebo effect, between the first and the third sessions the immediate effect of MPA, between the first and the fourth and the first and the fifth the sustained effect of MPA. Correlation analyses were performed with Pearson correlation method and p-values were corrected with Bonferroni method. In all tests, p < 0.05 was considered significant. Statistical computing was performed with SAS statistical package (version 8.01, SAS Institute, Cary, NC).

## Results

13 out of 15 patients completed the trial. Two subjects discontinued due to acute exacerbation of their COPD after the second visit. None of the subjects had clinical symptoms of OSAS. Mean (± SD) ODI_4 _was 1.4 ± 1.9/h at baseline and did not change during the trial. Laboratory assessments were within inclusion criteria in all but one patient. She had low serum FSH (S-FSH 25 IU/L). However, she was considered postmenopausal based on her age (71 years) and her serum concentration of estradiol (36 pmol/L, postmenopausal reference range < 140 pmol/L), and included in the trial. According to tablet count, all patients were compliant and after the two-week treatment MPA was detectable in all patients confirming their compliance. After a three-week washout MPA was under the detection limit in three patients and near the detection limit in the rest, and after a six-week washout undetectable in all patients. No correlations were found between serum MPA levels and SaO_2 _or tcCO_2_. We were not able to analyse the EtCO_2 _data due to insufficient plateaus in EtCO_2 _signals.

Baseline characteristics of patients are presented in Tables 1 and 2. Weight, dyspnea or other symptom scores, blood pressure, or spirometric values did not differ during the trial. None of the patients had a history of snoring, witnessed apnea or excessive sleepiness. Patients reported that they slept as usual and woke up several times during night. They had no specific complaints disturbing their sleep. Subjective sleep quality and total time in bed did not differ throughout the trial. MPA did not induce any alterations in objectively measured polygraphic sleep parameters. Three women had withdrawal bleeding after cessation of MPA therapy, one of them was on vaginal estrogen therapy. The other two women had benign endometrial findings underlying their withdrawal bleeding.

**Table 1:** Characteristics of patients at baseline.

**N = 15**	**Mean**	**SD**	**Range**
**Age (yr)**	67.5	6.0	56 – 76
**BMI (kg/m^2^)**	26.9	4.9	15.4 – 35.2
**Smoking (pack-year)**	8.8	13.9	0 – 50
			
**FEV_1_(L)**	0.76	0.3	0.44 – 1.80
**FEV_1_(%)**	34	12.4	15 – 63
**FVC (L)**	1.25	0.5	0.77 – 1.69
**FVC (%)**	44	16.2	26 – 72
**FEV%**	61	13.7	48 – 77
			
**Arterial pH**	7.38	0.05	7.25 – 7.45
**PaCO_2_(kPa)**	6.0	1.1	5.5 – 9.9 74.2
**PaO_2 _(kPa)**	9.0	1.2	5.6 – 12.6
			
**Systolic BP (mmHg)**	154	21.8	120 – 190
**Diastolic BP (mmHg)**	87	14.1	60 – 118

BMI = body mass index, FEV_1 _= forced expiratory volume in one second, FVC = forced vital capacity, FEV% = 100 × (FEV_1_/FVC), PaCO_2 _= partial tension of arterial carbon dioxide, PaO_2 _= partial tension of arterial oxygen.

**Table 2:** Objective sleep quality at baseline, N = 15.

**Sleep Recording Data at Baseline**	
TST	4 h 34 min ± 1 h 13 min
Sleep efficiency	66 ± 18 % of sleep period time
EEG arousals	2.6 ± 3.3 / h of sleep
Movement arousals	9.7 ± 7.1 / h of sleep
Latency to S2 sleep	41 ± 41 min
Stage S1 sleep	87 ± 60 min (34 ± 24 %)
Stage S2 sleep	81 ± 48 min (29 ± 15 %)
SWS	73 ± 39 min (26 ± 10 %)
REM sleep	34 ± 31 min (11 ± 10 %)

Percentages indicate proportion of the total sleep time (TST) unless otherwise indicated. Data presented as mean ± SD. REM = rapid eye movements, SWS = slow wave sleep (sleep stages 3 and 4), TST = total sleep time.

**Figure 2:**
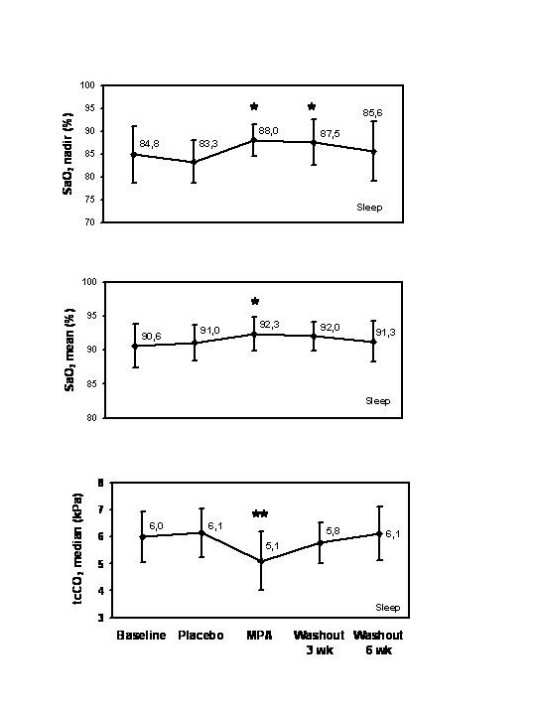
**Changes of SaO_2 _nadir, SaO_2 _mean, and tcCO_2 _median during sleep with MPA and after a 3- and a 6-week follow-up. **Changes are absolute percentage values. MPA = medroxyprogesterone acetate, SaO_2 _= arterial oxygen saturation, tcCO_2 _= transcutaneous partial carbon dioxide tension. The error bars of SaO_2 _mean and tcCO_2 _median represent standard deviation and those of SaO2 nadir represent interquartile ranges. P-values are corrected with Bonferroni method. ** = p < 0.01, * = p < 0.05.

No changes in respiratory or sleep parameters were found during placebo period compared to baseline. We analyzed the effects of MPA during sleep and separately according to sleep stages. Both SaO_2 _mean and SaO_2 _nadir improved in 11 out of 13 (85 %) patients during sleep. tcCO_2 _median improved with MPA in 12 out of the 13 women (92 %). At baseline, the SaO_2 _mean averaged 90.6 ± 3.2 % (95% Bonferroni corrected confidence interval (CI) 88.3, 92.9; Fig. 2). The average increase with MPA was 1.7 ± 1.6 %-units (95% CI 0.56, 2.84; p = 0.044). The median of SaO_2 _nadir was 84.8 % (interquartile range, IQR 6.1; 95% CI 66.5, 87.3) at baseline and improved by 3.9 %-units (IQR 4.8; 95% CI 0.24, 10.2; p = 0.024; Fig. 2.) with MPA. At baseline, the mean of tcCO_2 _median was 6.0 ± 0.9 kPa (CI 95% 5.4, 6.6) and decreased by 0.9 ± 0.5 kPa with MPA (CI 95 % -1.3, -0.5; p = 0.004; Fig. 2.) There was a borderline increase in the median of maximum inspiratory slope from 10.4 (IQR 4.7; 95% CI 7.0, 23.7) U/50-1 at baseline to 18.9 (IQR 22.4; CI 95% 11.0, 62.9) U/50-1 with MPA (p = 0.068).

Three weeks after cessation of MPA, SaO_2 _mean and SaO_2 _nadir sustained to be higher in 11 out of 13 patients during sleep, when compared to baseline. The average of SaO_2 _mean was 1.4 ± 1.8 (95% corrected CI -0.03, 2.8) %-units higher, the difference being statistically not significant after Bonferroni corrections (p = 0.028 prior to the correction; p = 0.112 after Bonferroni correction, n = 11, Fig. 2). The median SaO_2 _nadir was 2.7 %-units (IQR 4.9; 95% CI 0.06, 18.7) higher than at baseline (p = 0.032). Six weeks after MPA administration SaO_2 _nadir or SaO_2 _mean did not differ from baseline. Within three weeks after cessation of MPA, tcCO_2 _median was lower compared to baseline in 6 out of 13 patients but the mean of tcCO_2 _median had returned to baseline (Fig. 2).

**Figure 3:**
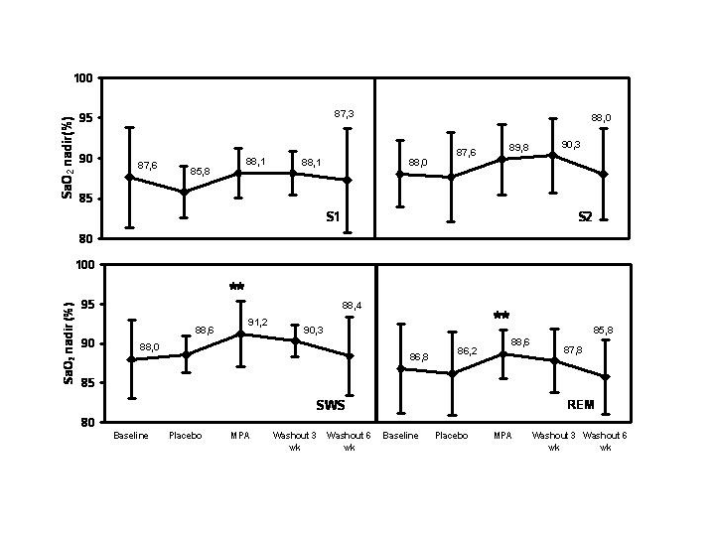
**Changes of SaO_2 _nadir during different sleep stages with MPA and after a 3- and a 6-week follow-up. **Changes are absolute percentage values. MPA = medroxyprogesterone acetate, SaO_2 _= arterial oxygen saturation. The error bars of SaO_2 _nadir represent interquartile ranges. P-values are corrected with Bonferroni method. ** = p < 0.01.

**Figure 4:**
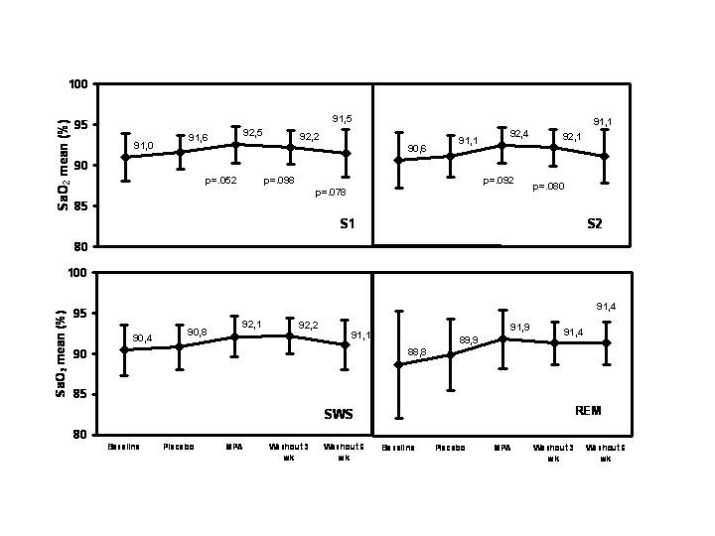
**Changes of SaO_2 _mean during different sleep stages with MPA and after a 3- and a 6-week follow-up. **Changes are absolute percentage values. MPA = medroxyprogesterone acetate, SaO_2 _= arterial oxygen saturation. The error bars of SaO_2 _mean represent standard deviation. P-values are corrected with Bonferroni method.

**Figure 5:**
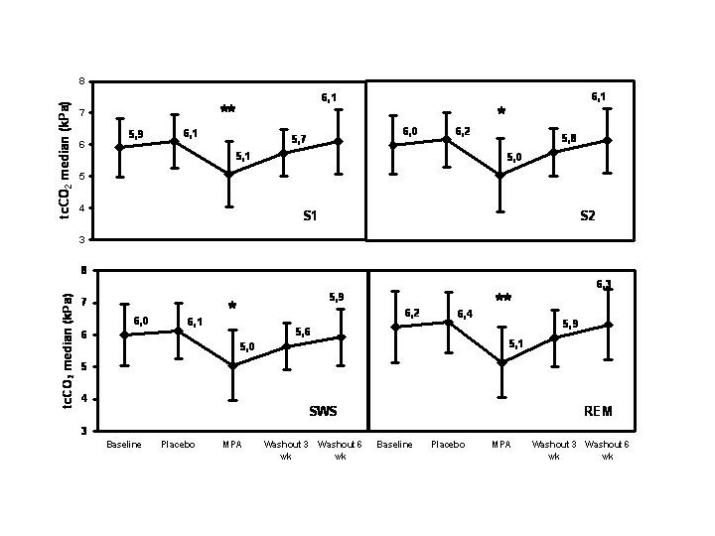
**Changes of the mean of tcCO_2 _median during different sleep stages with MPA and after a 3- and a 6-week follow-up. **Changes are absolute percentage values. MPA = medroxyprogesterone acetate, tcCO_2 _= transcutaneous partial carbon dioxide tension. The error bars of tcCO_2 _represent standard deviation. P-values are corrected with Bonferroni method. ** = p < 0.01, * = p < 0.05.

The pattern of MPA effect on SaO_2 _nadir (Fig. 3), SaO_2 _mean (Fig. 4), and tcCO_2 _median (Fig. 5) was quite similar during stages S1, S2, slow wave sleep (SWS; stages S3 and S4) and rapid eye movement (REM) sleep. Importantly, MPA had a marked effect also during REM sleep (Fig. 3, 4, 5). The median of maximum inspiratory slope increased from 10.7 (IQR 4.6; 95% CI 6.5, 30.0) U/50-1 at baseline to 16.8 (IQR 30.7) U/50-1 with MPA (95% CI 11.8, 59.0; p = 0.042) during stage 4 sleep.

Increases in SaO_2 _mean and SaO_2 _nadir during sleep with MPA were inversely associated with baseline SaO_2 _mean (r = -0.70, p = 0.032) and baseline SaO_2 _nadir (r = -0.77, p = 0.008), respectively. Decrease in tcCO2 with MPA did not correlate neither with baseline SaO_2 _nor tcCO_2 _levels. Treatment response in SaO_2 _mean, SaO_2 _nadir and tcCO_2 _levels did not associate with pack-years smoked, age, BMI, baseline spirometric results or sleep variables.

## Discussion

The present data show that MPA effectively improves SaO_2 _and tcCO_2 _during sleep in postmenopausal women with moderate to severe COPD. The improvement seems to be greater in patients with lower baseline SaO_2_. None of the previous studies has systemically used MPA to stimulate nocturnal breathing in female patients with COPD. The effect seems to be more consistent in female than in male patients with COPD[5]. The pattern of MPA effect was quite similar over the array of sleep stages and MPA had beneficial effects also during REM sleep. Even when administered at higher doses than in ordinary gynecologic indications, MPA was also well tolerated.

We used the maximum inspiratory slope as an index of respiratory drive. Although we have used the inspiratory slope index previously in patients with partial upper airway obstruction during sleep[19,20], it can also be used in populations without partial upper airway obstruction such as in patients with COPD. It is a non-invasive index affected also by upper airway dynamics. The different contributing factors are not calculated separately but the index reflects a "total output" during the initial part of inspiration. Since this index is insufficiently validated, the results should be considered suggestive and interpreted with caution. At baseline, the maximum slope in this group of women with moderate to severe COPD was compatible with that of healthy postmenopausal women[20]. It was also compatible with the maximum inspiratory slope in postmenopausal women with partial upper airway obstruction during sleep using MPA[20]. Although the maximum inspiratory slope was already at baseline within normal postmenopausal range, MPA almost doubled the slope. This finding is in line with the thinking that the baseline respiratory drive in women with COPD is not decreased compared to healthy postmenopausal women and that exogenous respiratory stimulation increases the respiratory drive above the physiological postmenopausal level in these patients.

Our results differ to some extent from the previous studies by other groups. First, in our female patients, the respiratory improvement seems to be more consistent than in the previous studies recruiting almost entirely male patients. SaO_2 _mean and SaO_2 _nadir increased in 11 out of 13 (85 %) and tcCO_2 _median decreased with MPA in 12 out of the 13 women (92 %) during sleep. Previously, in 17 men and two women with COPD, MPA (60 mg/d/1 month) improved SaO_2 _during sleep only in five out of 19 patients[5]. The authors did not report, whether both of the studied women were among the responders. In six non-obese men with COPD, MPA (100 mg/d/15 days) decreased the number of hypoxemic episodes during sleep[6].

Second, the CO_2 _levels were not monitored during sleep in most previous studies. In the present study, lowering of tcCO_2 _was short-lived paralleling to the pattern observed in base excess previously in awake patients[10]. Our findings are consistent with those observed in 17 hypercapnic stable patients with COPD, where nocturnal end-tidal CO_2 _decreased by 0.9 kPa with MPA (60 mg/d/2 weeks)[9]. Our results are also in line with observations in awake healthy males, where MPA increased alveolar ventilation and slopes of hypercapnic and hypoxic ventilatory responses[23]. tcCO_2 _reflects the metabolic tissue performance[24] and the slow changes of the absolute partial arterial carbon dioxide tension (PaCO_2_)[25]. According to Sanders and co-workers,[26] tcCO_2 _does not consistently and accurately reflect PaCO_2 _and may even overestimate it[27]. However, tcCO_2 _measurements show the direction and rough magnitude of the changes in CO_2 _levels. There is no indication that PaCO_2 _would be superior to tcCO_2 _to monitor the metabolic condition at the tissue level. We used tcCO_2 _as a surrogate for PaCO_2 _in order to avoid invasive measurements. Nothing suggests so far that transcutaneous CO_2 _measurements would affect sleep quality. However, we think that neither end tidal CO_2 _nor tcCO_2 _represents directly PaCO_2 _but that all the three measurements reflect different phenomena. Frequent nocturnal sampling of arterial blood gases would increase our understanding about the blood-tissue interactions of acid-base balance and needs to be considered in future studies.

Third, MPA effectively improved oxygenation and tcCO_2 _also during REM sleep. In a previous study in patients with COPD, MPA (60 mg/d/4 weeks) decreased PaCO_2 _and increased PaO_2 _only during non-REM sleep and had a similar trend during REM sleep[28].

Fourth, the possible after-effects of MPA on breathing were usually not evaluated in previous studies. The respiratory effects of a two-week treatment with MPA of 60 mg daily subsided within 14 days in healthy male subjects[29]. In patients with COPD, Wagenaar and coworkers did not observe any after-effects on respiration one month after cessation of combined therapy with MPA and acetazolamide (gender distribution in study population not reported)[9]. Prolonged improvement of daytime PaCO_2 _in women with chronic respiratory insufficiency[10] and of nocturnal end tidal CO_2 _in postmenopausal women with partial upper airway obstruction during sleep[13] has been a consistent finding in our previous studies. The present study showed a sustained improvement in SaO_2 _nadir.

The obviously more consistent and prolonged effect of MPA on respiration in our female patients compared to that in male patients in previous studies may be explained by differences in study populations or MPA dosage regimen. We recruited only women. Although both estrogen and progesterone concentrations in postmenopausal women are at the same level than in men, the effects of progestin therapy may remain gender-specific. Sex steroids influence neuromodulatory serotonergic neurons,[30] which are critically involved in the neural control of breathing[31,32]. Serotonin has an excitatory effect on upper airway and phrenic motoneurons [33-37]. Animal studies demonstrate a greater serotonin activity in female than in male brain[30,38]. The number and distribution of progesterone receptors may differ between genders because androgens down-regulate progesterone and estrogen receptors without affecting progesterone or estradiol concentrations[39]. Estradiol increases the number of progesterone receptors[40]. In ovariectomized rats, MPA did not stimulate breathing until progesterone receptors were upregulated with estrogen[41]. MPA-derived metabolites with intrinsic estrogenic activity[42] may upregulate progesterone receptors more effectively in women than in men.

The duration of respiratory effects of MPA may differ between healthy individuals and those with respiratory impairment like COPD or sleep-disordered breathing. The persistent effect of MPA may be due to modification of peripheral or central chemoreceptor action or central processing of the carotid body neural output[43]. MPA may reset the respiratory center for a new response level. The homeostatic regulatory mechanisms aim to maintain the normal function in the body. Therefore, the new response threshold of the respiratory center is maintained longer in subjects with respiratory impairment than in healthy subjects. The elimination of MPA might be slower in diseased patients than in healthy individuals. However, this is unlikely to explain the persistent respiratory effect in our patients, since MPA concentrations were below or near the detection limit within three weeks after cessation of MPA.

Our relatively high MPA dose was chosen to ensure effective respiratory stimulation during night. Therefore, we also administered the whole dose of MPA in the evening. MPA reaches its peak serum concentration within one to three hours and the concentrations decline quite rapidly thereafter[14]. 60 mg per day is the most commonly used dose in studies where respiration is stimulated by MPA. However, it is usually divided in three doses administered at 8-hour intervals. MPA-induced persistent respiratory effects may be attributed to the alteration in endocrinological environment, or pharmacodynamics of MPA. MPA alters hormone levels, and their recuperation may take for weeks at least in women[12]. In an animal model, the clearance of MPA-related substances is slow in the lung, skeletal muscle and brain tissues[44]. Although MPA has a stronger progestational activity than progesterone, it does not have greater effects on breathing than progesterone[29]. This observation may indicate that the metabolites of MPA cause the respiratory effects rather than MPA itself.

MPA did not have any effect on subjective or objective sleep quality in our patients. In postmenopausal women, MPA (5 mg/d) combined with estrogen had no effect on objective sleep parameters but improved subjective sleep quality[45]. According to previous studies, MPA has induced no consistent effects on sleep[5,6,13,28,46-50].

The arousal indexes were low. Part of this could be due to true suppression of arousals by hypoxemia and sleep deprivation. On the other hand, many of the arousals initiated a whole epoch of wakefulness, resulting in technically low arousal index, compared to healthy elderly people or patients with COPD.

Because our patients spent most of the time in light sleep, arousals easily resulted in awakenings and thereby in lower arousal index than previously reported in healthy elderly people[51] or in patients with COPD[52,53]. Our patients were also hypoxemic and sleep deprived with an average total sleep time less than 5 hours. Both hypoxemia[54,55] and sleep deprivation [56,57] increase arousal threshold resulting in lower arousal index.

The single blind study design is a limitation of our study. We refrained from using a cross-over design knowing that acute exacerbations frequently occur in patients with moderate to severe COPD, and therefore the longer duration of the study would increase the drop-out rate markedly. Although a cross-over setting was not feasible, a parallel group design would have strengthened the data and needs to be considered for future trials. Another limitation is the small study population. Although the number of patients recruited was based on our previous trials[10,13], the results of the current study indicated that a greater number of subjects would have strengthened especially the tcCO_2 _results. However, we are confident that correction of these shortcomings would only have been strengthened, not undone our findings.

## Conclusion

Taken together, MPA 60 mg daily for 14 days improved breathing during sleep in postmenopausal women with COPD. Some of the effects persisted for weeks after cessation of MPA. Our patients were not homogenous in terms of baseline arterial blood gases and nocturnal SaO_2_. Some of them had values likely to threaten their life expectancy, others had minor impairments. Patients with more severe hypoxemia seemed to improve more than those with milder respiratory impairment. Our results warrant further studies into the long-term efficacy and feasibility of MPA administered either on a cyclical or on a continuous basis to support breathing during sleep in postmenopausal women with clinically significant hypoxia or hypercapnia.

## List of abbreviations

ANOVA = analysis of variance, CI = confidence interval, COPD = chronic obstructive pulmonary disease, IQR = interquartile range, MPA = medroxyprogesterone acetate, FEV_1 _= forced expiratory volume in one second, FSH = follicle-stimulating hormone, SaO_2 _= arterial oxyhemoglobin saturation,

REM sleep = rapid eye movement sleep, SWS = slow wave sleep, tcCO_2 _= transcutaneous carbon dioxide tension

## Competing interests

The author(s) declare that they have no competing interests.

## Authors' contributions

TS participated in the design of the study, coordinated and carried out the studies, analyzed the sleep recordings, participated in the analyses and interpretation of data, and drafted the manuscript.

TA constructed the analysis model for tcCO_2 _signal, participated in the analyses and interpretation of tcCO_2 _data and in the statistical analyses, and drafted the manuscript.

KU participated in the analyses and interpretation of tcCO_2 _data, and drafted the manuscript.

OP conceived of the study, and participated in its design and in the interpretation of data, and drafted the manuscript. All authors read and approved the final manuscript.
